# Assessment of physical activity level in young adults with eating disorder risk: a cross-sectional study in a non-clinical sample

**DOI:** 10.1192/j.eurpsy.2022.1482

**Published:** 2022-09-01

**Authors:** M. Mróz, A. Brytek-Matera

**Affiliations:** University of Wroclaw, Institute Of Psychology, Wroclaw, Poland

**Keywords:** physical activity, eating disorder risk, non-clnical sample

## Abstract

**Introduction:**

Physical activity (PA) level has been found to be an important correlate of eating disorders (EDs). The literature is inconclusive to whether PA is related to symptoms of EDs in non-clinical sample.

**Objectives:**

The first study aim was to assess the level of PA in non-clinical group of young adults with symptoms of EDs. The second aim was to evaluate the association between PA level and severity of EDs symptoms.

**Methods:**

The sample consisted of 327 young adults (M_age_ = 21.72±2.00;M_BMI_ = 23.20±7.43). All participants completed the *Eating* Attitudes Test (*EAT-26*) and the *International Physical Activity Questionnaire*(IPAQ). Finally, 32 individuals (9.79%) of the total sample scored above clinical cut-off on the EAT-26 (≥ 20) indicating a high level of symptoms and concerns characteristic of EDs.

**Results:**

The non-clinical group differed significantly in PA level (low-intensity, moderate-intensity, vigorous-intensity levels of PA; H(2,32)=26.19,p<0.001). There was no difference in the severity of ED symptoms between the groups of PA level. Our findings demonstrated a positive relationship between PA (IPAQ total score) and bulimic behaviour and thoughts about food (rho-Spearman=0.31,p=0.04). The highest Bulimia and Preoccupation scale scores were observed in group with vigorous-intensity levels of PA (Me=8.5).

**Conclusions:**

Our findings indicate that the severity of ED symptoms did not differ across the PA levels in a non-clinical sample of young adults. However, PA was positively associated with bulimia and food preoccupation. Since, excessive physical could be an important risk-factor of EDs, the recommended levels of PA for health in non-clinical sample should be enhanced in effective prevention programs.

**Disclosure:**

No significant relationships.

